# Early Sympathy and Social Acceptance Predict the Development of Sharing in Children

**DOI:** 10.1371/journal.pone.0052017

**Published:** 2012-12-13

**Authors:** Tina Malti, Michaela Gummerum, Monika Keller, Maria Paula Chaparro, Marlis Buchmann

**Affiliations:** 1 Department of Psychology, University of Toronto, Mississauga, Ontario, Canada; 2 Department of Psychology, University of Plymouth, Plymouth, England, United Kingdom; 3 Max Planck Institute for Human Development, Berlin, Germany; 4 Jacobs Center for Productive Youth Development, University of Zurich, Zurich, Switzerland; Ecole Normale Supérieure, France

## Abstract

Sharing is a fascinating activity of the human species and an important basis for the development of fairness, care, and cooperation in human social interaction. Economic research has proposed that sharing, or the willingness to sacrifice own resources for others, has its roots in social emotions such as sympathy. However, only few cross-sectional experiments have investigated children’s other-regarding preferences, and the question how social-emotional skills influence the willingness to share valuable resources has not been tested. In the present longitudinal-experimental study, a sample of 175 6-year-old children, their primary caregivers, and their teachers is examined over a 3-year period of time. Data are analyzed by means of growth curve modeling. The findings show that sharing valuable resources strongly increases in children from 6 to 9 years of age. Increases in sharing behavior are associated with the early-developing ability to sympathize with anonymous others. Sharing at 7 years of age is predicted by feelings of social acceptance at 6 years of age. These findings hold after controlling for children’s IQ and SES. Girls share more equally than boys at 6 and 7 years of age, however, this gender difference disappears at the age of 9 years. These results indicate that human sharing strongly increases in middle childhood and, that this increase is associated with sympathy towards anonymous others and with feelings of social acceptance. Additionally, sharing develops earlier in girls than in boys. This developmental perspective contributes to new evidence on change in sharing and its social-emotional roots. A better understanding of the factors underlying differences in the development of sharing and pro-social orientations should also provide insights into the development of atypical, anti-social orientations which exhibit social-emotional differences such as aggression and bullying behavior.

## Introduction

Sharing is a fascinating activity of the human species and a focus of interest in various disciplines including psychology, economics, and evolutionary science. It exemplifies the willingness to take the welfare of others into account and thus represents “other-regarding” preferences [1]. Investigating the developmental antecedents of such “other-regarding” preferences will ultimately help in understanding the roots of fairness, caring, and cooperation in human social interaction [1], [2]. Here we use sharing resources with anonymous others as an empirical indicator of “other-regarding” preferences [1] and one subtype of prosocial behavior.

Prosocial behaviors have been studied by psychologists for decades [3]. Most of these studies have focused on other forms of prosocial behavior, such as children’s instrumental or altruistic helping or providing emotional support for needy others. These behaviors are either measured experimentally [4], [5] or assessed through observations [6], parent reports or teacher reports [7], [8]. More recently the behavioral economics approach of evaluating sharing with anonymous others, such as the Dictator Game, has become an interdisciplinary paradigm to study other-regarding preferences [9], [10]. Sharing resources with anonymous others, like other forms of prosocial behavior (e.g., instrumental or altruistic helping), is based on a concern for others’ needs and goals and the motivation to assist them [11]. Yet, sharing resources with anonymous others in the Dictator Game incurs real tangible costs for the actor, whereas the efforts associated with measures of prosocial behavior are generally low-cost (see [12]). Furthermore, whereas many experimental, observational, and questionnaire measures of prosocial behavior cannot be easily operationalized for different age groups, the strength of using the behavioral economic paradigm lies in the fact that the same experimental instrument (i.e., the Dictator Game) can be used across wide range of age groups which maximizes the ability to draw meaningful comparisons across development [13]. Because proposers in a one-shot dictator game only interact once with an anonymous other player who cannot reciprocate or punish in a future round of the game, their positive offers have been interpreted as altruistic or have been attributed to their fairness concerns [13].

Only a few experiments have investigated children’s other-regarding preferences in this paradigm [9], [10], [14], [15] , and whether younger children are self-serving or prefer equality in resource allocations is still debated [10], [16] . A recent experiment examining sharing has shown that 7- to 8-year-olds, but not 3- to 4-year olds, prefer equal resource allocations when sharing with friends and acquaintances [1]. There have not been any empirical studies that have examined the social-emotional antecedents of children’s sharing resources with anonymous others.

All of the studies that assessed sharing resources with behavioral economic tasks, such as the Dictator Game, have relied on cross-sectional data sets composed of children who vary in age. Thus, although there is some limited evidence that children’s other-regarding preferences increase from early-to-middle childhood, it is not known if this developmental increase applies to all children equally or if some children have these preferences from early on. If the latter is true, it remains an open and intriguing question in what characteristics children with early-existing other-regarding preferences might differ from children who develop these preferences in middle childhood.

In order to measure how children’s willingness to share resources with others develops over time, and to understand which factors influence these other-regarding preferences, longitudinal data sets are necessary [17]. In order to assess individual stability of sharing across development we investigated 175 Swiss children (85 girls) in a longitudinal-experimental study. The children were assessed at 6 years of age, 7 years of age, and 9 years of age with the Dictator Game, a paradigmatic economics task of prosocial sharing behavior with an anonymous other (see below).

We focused on two socio-emotional antecedents of sharing, namely sympathy and social acceptance. The question of whether children’s other-regarding preferences are rooted in sympathy is striking, as recent experiments suggest that non-human primates are able to sympathize with others, especially if these others are members of their immediate social group [18], [19]. Such in-group preference has also been observed in preschool-children [20].

Sympathy entails feelings of concern for the other person based on an understanding of that person’s circumstances [21], [22]. Sympathy (i.e., other-oriented concern), like empathy (i.e., emotional contagion), involves the comprehension or apprehension of another’s affective state. Unlike empathy, however, sympathy primarily entails other-oriented concern and not the experience of the same or a similar emotion as the other [23]. In this way, sympathy entails a degree of distancing between the self and the other that is not present in empathy [21]. Sympathy has been posited by theorists to be an important motive of morally relevant, prosocial behavior [24], and it might be an antecedent of sharing resources with anonymous others [25]. In contrast, empathy might not lead to prosocial behavior, because it can either lead to sympathy for another or personal distress. Thus, empathy would relate positively to prosocial behavior only in specific situations [26]. In addition, empathic overarousal can lead to feelings of being overwhelmed so that one cannot be concerned with the needy other [22].

We investigated whether developmental processes in sharing with anonymous others depend on the earlier propensity to sympathize with anonymous others who are not members of the immediate social group. The focus on the early social-emotional roots of humans’ other-regarding preferences is new and fascinating because philosophers and psychologists have argued that social emotions play a role in the development of prosocial behaviors and are important motivators for prosociality in general [21]. Previous psychological and economic research has mostly focused on the cognitive antecedents of other-regarding preferences, such as theory of mind.

Sharing may not only be influenced by the capacity to sympathize with anonymous others, but also by the extent to which one feels socially accepted by others, especially peers [27], [28]. Peer acceptance is important to children's social, emotional, and behavioral development, because it provides opportunities to learn and interact with children, which in turn promotes development [29]. Humans need to feel a sense of acceptance and belonging to develop an other-orientation [30]. We examined if sharing valuable resources with others relies on early feelings of being accepted by peers; does a child’s need for acceptance predict how they will subsequently share with anonymous others?

Sharing seems also to differ across gender. There is evidence that females are more averse to unequal sharing than males [31]. Yet, it is not known when these gender differences in other-regarding preferences emerge in humans. The few existing findings on early gender differences in sharing are inconsistent. Some studies show no differences, and others show that females share more generously than males in some age groups [32], [33]. If indeed there are evolutionarily-derived gender differences in sharing, then it is likely that they evolve early in life.

We studied children’s sharing when they were 6, 7 and 9 years of age. According to Fehr et al. [1], at the age of 6, unequal, self-serving distributions should dominate, whereas by the age of 9, about half, or more, of the participants should show equal allocations. At each of the three time points, sharing behavior was assessed in a one-shot experiment with anonymous interaction partners [8], [9], [10], [33]. We presented children with identical stickers and asked them to distribute the stickers, in any way they want among themselves and an anonymous child of the same age and gender (see Materials and Methods). Via standardized questionnaires, we asked primary caregivers to report on their children’s sympathy towards anonymous distressed others at each time point, as well as on their children’s feelings of social acceptance. We also obtained these reports from the children themselves and from the children’s classroom teachers through the same standardized questionnaire at each time point. Multi-informant measures have been shown to be the most reliable sources of information on children’s social-emotional skills [34]. In order to control for variables known to be of influence on other-regarding preferences [3], we collected information about the family’s socioeconomic status (SES) and obtained the children’s intelligence quotient.

## Results

The descriptive statistics of all study variables, as well as the correlation coefficients across all main study variables, are presented in Table 1.

**Table 1:** Means (standard deviations), ranges and Spearman correlation coefficients of the main study variables.

	*M(SD)*	Range	1	2	3	4	5	6	7	8	9	10	11
1. Sharing at T1 (Age 6)^a^	.44(.16)	0–1											
2. Sharing at T2 (Age 7)^a^	.47(.10)	0–.83	.15*										
3. Sharing at T3 (Age 9)^a^	.48(.07)	0–.92	.09	.12									
4. Sympathy at T1 (Age 6)	.54(.16)	0–.92	.04	.11	.04								
5. Sympathy at T2 (Age 7)	.61(.13)	.17–.92	.13	.14	.12	.23**							
6. Sympathy at T3 (Age 9)	.68(.14)	.22–1	.15	.23**	.17*	.29**	.48**						
7. Social acceptance at T1 (Age 6)	4.14(.65)	1.96–6	.01	.12	.18*	.26**	.21**	.15					
8. Social acceptance at T2 (Age 7)	4.17(.71)	2.13–6	.10	.00	-.02	.10	.39**	.22**	.51**				
9. Social acceptance at T3 (Age 9)	4.04(.90)	1–6	.20**	.04	-.11	.09	.30**	.36**	.41**	.56**			
10. Intelligence quotient (T1)	97.1(11.71)	74–132	.05	.05	.01	.22**	.07	.13	.22**	.11	.05		
11. Socioeconomic status (T1)	5.78(2.45)	1–10	-.09	.08	.09	.14	.07	.15	.16*	.09	.07	.22**	
12. Gender (T1)	-	-	.17*	.18*	.20**	.17*	.27**	.26**	.17*	.14	.19*	.03	.07

** *p*<.01, *p<.05,

a Sharing scores represent proportional scores.

*Notes.* T1 = Time 1. T2 = Time 2. T3 = Time 3.

**Figure 1:**
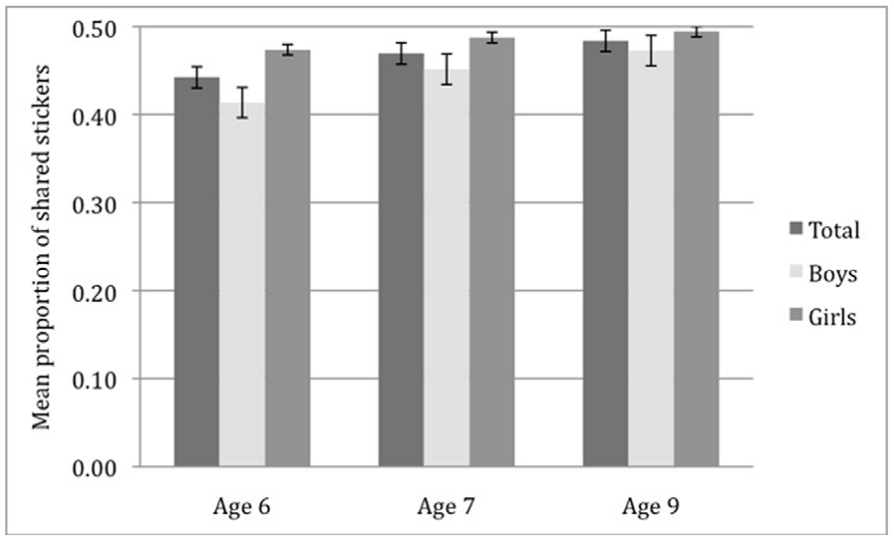
Development trend of children’s sharing from 6 to 9 years as a function of gender. The figure shows mean proportion scores of shared stickers as a function of age and gender (N = 175). Growth curve modeling indicates that there is a significant increase in sharing over time, Intercept Est. = 0.45, *SE* = 0.01, *p*<.001, and Slope Est. = 0.01, *SE* = 0.01, *p*<.01. Proportions of shared stickers at each time point are calculated separately for boys and girls and compared using one-way ANOVAs. At Time 1 (age 6) and Time 2 (age 7), girls share significantly more stickers than boys, *F*(1, 174) = 6.00, *p* = .02 , η2 = .03, and *F*(1, 174) = 6.07, *p* = .02, η2 = .03, respectively. At Time 3 (Age 9), boys catch up, and the difference between the number of stickers shared by girls and boys is not significant, *F*(1, 174) = 2.65, *p* = .11. Error bars represent *SEM*; *p<.05.

Overall, 43% of the children shared evenly across all assessment points. Fifty-seven percent of the children were not willing to equally allocate resources at least one time point. We used unconditional latent growth modeling in Mplus (version 6.11) to test if there was growth in sharing, and if there was variability among the children in their growth curves [35]. The unconditional linear growth model provided good fit to the data, *AIC* = −881.06, *BIC* = −862.07. Both the intercept and slope factors were significant for the sharing model, Intercept Est. = 0.45, *SE* = 0.01, *p*<.001, and Slope Est. = 0.01, *SE* = 0.01, *p*<.01, respectively. We found an increase in children’s sharing from the age of 6 to 9 years (see Figure 1), and there was significant variability among children in the intercept and linear slope.

We found a strong gender effect in sharing. On average, girls shared more than boys at 6 years of age, *F*(1, 174) = 6.20, *p* = .01 , η2 = .04, and at 7 years of age, *F*(1, 174) = 6.02, *p* = .02, η2 = .03. In contrast, girls and boys did not differ in the number of stickers shared when they were 9 years of age, *F*(1, 174) = 3.41, *ns* (see Figure 1).

Next, to test our hypothesis regarding the effects of sympathy and social acceptance on the development of sharing, we estimated two latent growth curve models with time-varying and time-invariant covariates. The time-varying covariate was matched to the later outcome. In other words, sympathy and social acceptance at 6 years of age was matched to sharing at 7 years of age, and sympathy and social acceptance at 7 years of age was matched to sharing at 9 years of age. The time-varying covariates were estimated to have a direct effect on the later-time sharing indicator [8]. Gender, verbal intelligence, and SES were added as time-invariant covariates and were used to predict the intercept and slope factors in the models.

The model for sympathy and sharing fit the data very well (Table 2). There were time-specific effects of sympathy at Time 1 on sharing at Time 2 (*p*<.01) and sympathy at Time 2 on sharing at Time 3 (*p*<.05), indicating that sympathy at Time 1 and Time 2 predicted an increase in levels of sharing at subsequent time points. This finding held even after controlling for gender, child intelligence, and SES. The model also indicated that after adding the time-varying sympathy variables, the initially significant time effect (i.e., increase in sharing with age) remained significant. This finding indicates that sympathy at Time 1 and Time 2 predicted growth above and beyond the trajectory captured by the growth factor; however, it did not fully account for the general increase in sharing with age. Gender predicted initial level of sharing; that is, girls showed higher initial levels of sharing than boys. Gender did not predict growth.

**Table 2:** Parameter estimates (standard errors) for the latent growth curve models with time-varying covariates for effects of sympathy on the development of sharing.

	Sharing
Mean intercept	0.29 (0.07)***
Mean slope	0.15 (0.03)***
Intercept variance	0.00 (0.00)
Slope variance	0.01 (0.00)
Intercept/ slope covariance	−0.36 (.10)***
Time-varying covariates	
Sympathy T1 - SharingT2	0.06 (0.02)**
Sympathy T2 - Sharing T3	0.08 (0.04)*
Time-invariant covariates	
Gender at T1^a^	0.05 (0.02)**
Verbal intelligence at T1^a^	0.00 (0.01)
SES at T1^a^	−0.01 (0.00)
Model fit	
χ^2^/df	8.58/9
RMSEA	0.00
SRMR	0.03

*Notes.* ^a^ Coefficients for the time-invariant covariates are reported for the intercept only. None of the covariates showed significant slope effects.

T1 = Time 1. T2 = Time 2. T3 = Time 3.

*** *p*<.001, **p<.01, *p<.05,

In contrast, the latent growth curve analysis yielded no significant effects of social acceptance on the increase in sharing. However, a regression analysis indicated that sharing at Time 2 was predicted by social acceptance at Time 1 (β = .16, *p*<.05). This finding held even after controlling for sharing at Time 1, gender, child intelligence, and SES (*R* ^2^ = .07, *F*(5, 174) = 2.43, *p*<.05). Sharing at Time 3 was not significantly predicted by earlier social acceptance (see Table 3).

**Table 3:** Results of the hierarchical linear regression analyses predicting sharing at Time 2 and Time 3 by earlier social acceptance.

Sharing Time 2			Sharing Time 3		
Independent variables	β	*R* ^2^ / *F* for step	Independent variables	β	*R* ^2^ / *F* for step
Step 1^a^		.04/ 2.27	Step 1^a^		.03/ 1.54
Gender	.18*		Gender	.13	
IQ	.06		IQ	−.04	
SES	.03		SES	.08	
Step 2		.07/ 2.43*	Step 2		.07/ 1.83
Social acceptance T1	.16*		Social acceptance T1/T2	.18*/-.10	
Sharing T1	.08		Sharing T1/T2	.12/-.13	

*Notes.* ^a^ Control variables.

T1 = Time 1. T2 = Time 2. T3 = Time 3.

* *p*<.05,

## Discussion and Conclusions

Given the pivotal role that other-regarding preferences play in fairness, caring, and cooperation, it is important to understand how they develop in humans [36]. Sharing has been regarded as an important indicator of other-regarding preferences. Notably, we found that children share more, and thus become less self-focused and more other-oriented, from 6 to 9 years of age. Studies have shown that instrumental helping (i.e., helping another individual achieve its instrumental goal) develops in early childhood [5], that young children share after having worked together to earn a reward [37], and that 7- to 8-year-olds, but not 3- to 4-year olds, prefer equal resource allocations when sharing with friends [1].

Our findings document that sharing equally with anonymous others increases from middle to late childhood. In addition, we show that for a substantial number of children, other-regarding preferences seem to exist from the age of 6 and remain highly stable across middle childhood. For others, however, social-emotional factors (i.e., sympathy, social acceptance) might play a central role in developing other-regarding preferences. The developmental process in other-regarding preferences is likely due to children’s growing concern with norms of fairness and caring [38], [39], [40]. Additionally, as children move from middle to late childhood, they may also learn that fairness and caring help them in earning respect and acceptance by their peers [29]. The latter may be the reason why they increasingly share resources with others.

Whether young children show either selfish or other-regarding preferences from early on may be due to differences in sympathy: Sympathy towards anonymous distressed others strongly predicted subsequent sharing, even after controlling for earlier sharing, intelligence, and family SES. These findings implicate that human sharing is critically shaped by the earlier propensity to sympathize with anonymous others. Evolutionary theories suggest that one of the driving forces behind altruism is sympathy, especially if members of one’s immediate social groups are involved [41]. In the present study, we demonstrated that concern for anonymous others in distress might be an important human capacity which leads to other-regarding preferences. According to psychological theories, sympathy is important for prosocial behavior, as feeling negative emotions when someone else is experiencing distress increases the likelihood of caring [24]. It is likely that these feelings result, in part, from processes of understanding others’ emotions (i.e. affective perspective-taking skills). Since perspective-taking skills develop over the course of middle childhood, children may increasingly care about others’ feelings and, as a result, act more prosocially towards them [38], [42].

The development of other-regarding preferences might also be due to differences in social acceptance. Our findings indicate that social acceptance at 6 years of age strongly predicted sharing at 7 years of age, even after controlling for earlier sharing, intelligence, and family SES. These findings are striking as they implicate that children’s sharing is critically shaped by whether they feel accepted by others earlier on. Psychological theories emphasize the need for belonging as one of the core human needs [27], [28]. Our findings provide evidence for the notion that being accepted by others at the kindergarten age leads to increased willingness to share valuable resources with others at the elementary-school age. This latter finding suggests that feelings of being socially accepted during the transition from kindergarten to elementary school, a time when children have to adjust to a new environment and new social groups, are particularly important for the development of other-regarding preferences.

Our results showed that important gender differences exist with respect to other-regarding preferences. Younger girls tended to share more than boys, but this gender difference disappeared when children got older. Thus, it seems that gender may play an important role in the emergence of sharing [43] in early and middle childhood. These results can be interpreted in different ways: Socialization theories assume that children become increasingly aware of their social reputation when they move from middle to late childhood [29]. Hence, both boys and girls may increasingly try to maintain a positive peer reputation by behaving prosocially [29].

Studies in the behavioural economic paradigm have usually assessed age-differences in other-regarding preferences among cross-sectional samples. Compared to cross-sectional studies, longitudinal data sets, such as the one analyzed in the current study, offer the advantage of assessing individual stability of sharing resources across development. Other psychological research investigated the development of prosocial behavior and related socio-emotional abilities longitudinally in toddlers and preschool children. For example, Zahn-Waxler and colleauges (1992) found that some aspects of sympathy, such as expressing concern for a person hurting her finger and attempts to comprehend the distress of this person, increased significantly in children assessed at 14 and 20 months of age, but that the frequency of prosocial acts (i.e., helping or comforting the distressed person) remained stable [4]. Knafo and Plomin (2006) showed that parents rated their children as significantly more prosocial at age 3 than at age 2 [7]. Taken together, these studies indicate that, beginning in toddlerhood, prosocial behavior increases over middle to later childhood. Further longitudinal studies should assess sharing with the same instrument over a wide age range (from toddlerhood to adolescence).

Our results revealed that the ability to sympathize with others and the feeling of being socially accepted were longitudinally predictive of increased sharing, but no cross-sectional relations were found in the multivariate analyses. These results suggest that sympathy and social acceptance play an important role in children’s early orientation towards the needs of others -an orientation that predicts generous sharing behavior later in development. The surprising finding that sympathy predicts later, but not concurrent, increases in sharing, may be due to the difference in salience that competing motivations to share or not share (e.g., motivations based on concerns of fairness or morality versus motivations based on hedonism) have at different stages of development. Existing research documents cross-sectional relations between sharing valuable resources with sympathy in 4-year-olds only, but not in 8- and 12-year-olds ( [23], see also [44]). Research also demonstrates that hedonistic concerns (i.e., a focus on obtaining desired outcomes for the self) exert a powerful influence over prosocial dilemmas, including decisions to share or not share, in early childhood [45], [46]. Prosocial decisions later in childhood, however, increasingly incorporate more differentiated concerns including those of fairness and morality, as well as concerns over reciprocity, need, merit, and social reputation [15], [23]. It may be the case that sympathy at the age of 6, though not salient enough to overcome hedonistic motivations to keep resources for the self, may predispose children to consider the needs and feelings of others in the face of competing concerns later in development. Similarly, social acceptance at the age of 6 may predispose children to share valuable resources later in development because being accepted by peers early in development may create subsequent trust in others, which has been shown to lead to an increase in sharing from early childhood to adulthood [47], and which is related to a more pro-social and less antisocial orientation towards peers later in development [48]. However, the effects for the findings on social acceptance were very small in size. Thus, these interpretations admittedly have to remain speculative. Clearly, future research is warranted to validate our findings.

In conclusion, the fact that children increasingly shared valuable resources with others shows that human children strongly develop other-regarding preferences from middle to late childhood. These preferences develop earlier in girls than in boys, but there are no gender differences in other-regarding preferences by late childhood and early adolescence [10]. Our finding regarding sharing being predicted by the early ability to sympathize with anonymous others demonstrates that sharing may be rooted in a human tendency to feel for others who are suffering, even if they are strangers. In addition, children’s sharing was in part driven by feelings of social acceptance, which indicates that an orientation towards others may also depend on feeling happy and safe in the company of others. These findings have important implications for clinical interventions aimed at increasing an orientation towards others and at decreasing antisocial behavior in children.

## Materials and Methods

### Ethics Statement

The current study consisted of non-invasive and unconstrained child interviews ; these interviews were conducted in separate rooms at schools and at home. According to the current regulations in the canton of Zurich in Switzerland (the so-called “Regulations of the Ethics Commission for Psychological Research”, 2011), there is no requirement for an ethics committee approval. According to this regulation (Article 5, paragraph 1), this study is exempted from requiring formal ethical approval. The study fully complies with the ethics guidelines given by this legal regulation (see Article 8, paragraph 2). The regulation is based on the “Ethical Principals of Psychologists and Code of Conduct” (as outlined in the so-called “Ethical Guidelines for Psychologists of the Swiss Society for Psychology, as amended on October 13, 2003) and the ethical standards of the American Psychological Association (APA). Only children for whom parental written informed consent was obtained participated in the study. The interviews began after receiving permission from the schools. The data were analyzed anonymously.

### Participants

The data were taken from the first three waves of a Swiss longitudinal study concerning social development from childhood to adolescence. A random sample of kindergarten children and their primary caregivers was drawn from residents of the canton of Zurich in Switzerland. Written informed consent was obtained from the primary caregivers. Interviews were conducted at T1 with 175 children and 175 primary caregivers. One-hundred and sixty-three of the primary caregivers (93%) filled in a supplementary questionnaire. The children had an average age of 6.10 years (*SD* = 0.19). There were 85 girls (48.6%) and 90 boys (51.4%). Of the primary caregivers, 98% gave written consent to contact the child’s kindergarten teacher, and 133 of the corresponding kindergarten teachers filled in a questionnaire (77%). The great majority of participants were White. At the second assessment (T2), interviews were carried out with 158 children; one child refused to participate and one mother refused to let the child participate because the child was too shy.

Consent to contact the teacher at T2 was obtained from 154 parents (96%), and 140 teachers (91%) filled in a questionnaire. At T2, the children had an average age of 7.08 years (*SD* = 0.20).

At the third assessment (T3; two years after T2), 141 interviews and 139 interviews were carried out with children and primary caregivers, respectively. One hundred and thirty-four (96%) of the primary caregivers completed a supplementary questionnaire that measured the child’s social development and the primary caregiver’s parenting style. Consent to contact the teacher at T3 was obtained from 141 parents (100%), and 130 teachers (93%) filled in a questionnaire. The average age of the children at T3 was 9.17 (*SD* = 0.21).

Sample attrition effects were tested by comparing the primary caregivers at T1 (*N* = 175) whose children dropped out with those whose children dropped out at T2 (N = 15) and T3 (N = 21) on demographic variables (i.e., highest primary caregiver education, marital status) and the study variables at T1. Children who dropped out at T2 had caregivers who were more likely to lack a significant other (25% of the caregivers were single) than children who stayed in the sample (7% of the caregivers were single) at T2, *χ^2^*(1, 175) = 4,44, *p* < .05. No other variables were related to attrition status. Although retention in the study was high, there were some missing data. Therefore, single imputation was carried out to estimate the values for the missing data points using the expectation maximization method (SPSS Version 19).

### Procedure

The first assessment was conducted during the spring of 2006. The second and third assessments were completed 1 and 2 years later respectively, using the same procedure as the one used at T1. There were three sessions for each child at T1, each lasting approximately 60 minutes: one at home consisting of a computer-assisted personal interview (CAPI) and video recording (observation) of the child’s interaction with the primary caregiver, and two sessions in quiet rooms at the kindergarten or school) using paper-and-pencil tests and video recording. The interviewers were undergraduate psychology students who had been intensively trained in the relevant interview techniques.

### Measures

#### Sharing behaviour

At all assessment points, spontaneous sharing behavior was measured by using the dictator game, a sharing task developed in experimental economics [9]. In the present study, participants had to share six identical stickers between themselves and another anonymous child of the same age and gender at T1 and T2. At T3, they shared twelve identical stickers. For data analysis, proportional scores were created by computing the number of shared stickers divided by the total number of stickers.

#### Sympathy

At all assessment points, children’s sympathy was assessed by (a) teachers’ ratings, (b) mothers’ ratings, and (c) self-ratings [49].

At T1–T3, the teachers and mothers rated the child’s sympathy on five items [49] using a six-point scale. For teacher-rated sympathy, Cronbach’s *α* = .92 at T1, .90 at T2, and .97 at T3. For mother-rated sympathy, Cronbach’s *α* = .83 at T1, .85 at T2, and .88 at T3.

At T1–T3, children rated their sympathy on a scale containing five items (from [49]; e.g., “When I see another child who is hurt or upset, I feel sorry for him or her”). The children were asked whether the sentence was like him/her or not, and, if so, how much (0 = *not like him/her*; 1 = *sort of like him/her*; 2 = *like him/her*). Cronbach’s α for the sympathy scale was .67 at T1, .73 at T2, and .74 at T3.

#### Social acceptance

At all assessment points, children’s social acceptance was assessed by (a) teachers’ ratings, (b) mothers’ ratings, and (c) self-ratings [50], [51], [52].

At T1–T3, the teachers and mothers rated the child’s social acceptance on five items using a six-point scale. The items were taken from the Strengths and Difficulties Questionnaire and a questionnaire on peer relations [50], [51]. For teacher-rated social acceptance, Cronbach’s *α* = .79 at T1, .82 at T2, and .87 at T3. For mother-rated social acceptance, Cronbach’s *α* = .75 at T1, .80 at T2, and .72 at T3.

At T1–T3, children rated their social acceptance on six items of the Pictorial Scale of Perceived Competence and Social Acceptance (from [52]; e.g., “This boy has friends to play with, and this boy has no friends to play with. Which boy are you more like?”). The children were asked to report which child they were more like, and the degree to which they were like the child in the picture (*sort of true for me; really true for me).* Thus, items were scored on a 4-point scale. Cronbach’s α for the social acceptance scale was .69 at T1, .85 at T2, and .60 at T3.

Because the primary caregivers’, teachers’, and children’s ratings of sympathy were predominantly significantly associated with each other at each time point, and to reduce the number of measures and increase reliability [53], they were averaged into overall scales labelled “sympathy at T1”, “sympathy at T2”, and “sympathy at T3”. The same was done for the primary caregivers’, teachers’, and children’s ratings of social acceptance, and the overall scales were labeled “social acceptance at T1”, “social acceptance at T2”, and “social acceptance at T3”.

#### Intelligence quotient

The children’s intelligence was measured at T1 using the “verbal intelligence” section of the German version of the Hamburg-Wechsler Intelligence Test (HAWIK-III).

#### Family socioeconomic status

For socioeconomic background, we coded both the primary caregivers’ and their partners’ highest educational attainment. Responses were coded 1 (primary or lower secondary education), 2 (vocational training), 3 (vocational college), 4 (baccalaureate degree or higher vocational diploma), and 5 (university degree). Education scores, which served as an index of socioeconomic status (SES), were then computed. Higher scores indicated higher SES.
